# Seasonal Variation in Physical Activity among Preoperative Patients with Lung Cancer Determined Using a Wearable Device

**DOI:** 10.3390/jcm9020349

**Published:** 2020-01-27

**Authors:** Sunga Kong, Hye Yun Park, Danbee Kang, Jae Kyung Lee, Genehee Lee, O Jung Kwon, Young Mog Shim, Jae Ill Zo, Juhee Cho

**Affiliations:** 1 Department of Clinical Research Design and Evaluation, SAIHST, Sungkyunkwan University, Seoul 06351, Korea; sunga00kong@gmail.com (S.K.); dbee.kang@gmail.com (D.K.); 2 Patient-Centered Outcomes Research Institute, Samsung Medical Center, Seoul 06351, Korea; roemgirls1230@gmail.com (J.K.L.); genehee@gmail.com (G.L.); 3 Division of Pulmonary and Critical Care Medicine, Department of Medicine, Samsung Medical Center, Sungkyunkwan University School of Medicine, Seoul 06351, Korea; hyeyunpark@skku.edu (H.Y.P.); ojung.kwon@samsung.com (O.J.K.); 4 Center for Clinical Epidemiology, Samsung Medical Center, Sungkyunkwan University School of Medicine, Seoul 06351, Korea; 5 Department of Thoracic and Cardiovascular Surgery, Samsung Medical Center, Sungkyunkwan University School of Medicine, Seoul 06351, Korea; youngmog.shim@samsung.com; 6 Departments of Epidemiology and Health, Behavior and Society, Johns Hopkins Bloomberg School of Public Health, Baltimore, MD 21205, USA

**Keywords:** lung cancer, physical activity, season, preoperative, wearable

## Abstract

We aim to examine how season and temperature levels affect physical activity using a wearable device among patients scheduled to undergo surgical resection of lung cancer. Physical activity (PA) data from the wearable device were analyzed by seasons for 555 preoperative lung cancer patients from the CATCH-LUNG cohort study. The seasons were divided into spring, summer, autumn, and winter using the study enrollment date before surgery. The overall mean (SD) age was 61.1 (8.9) years, and the mean (SD) daily steps at each season were 11,438 (5922), 11,147 (5065), 10,404 (4403), and 8548 (4293), respectively. In the fully-adjusted models, patients in the winter season had 27.04% fewer daily steps (95% CI = −36.68%, −15.93%) and 35.22% less time spent performing moderate to vigorous physical activity (MVPA) compared to patients in the spring. The proportion of participants with over 8000 steps and duration of MVPA were significantly lower in the winter than the spring. In particular, daily steps had a negative linear association with wind chill temperature in patients who lived in Seoul. In conclusion, PA was significantly lower in the winter and it was more robust in patients who had a low cardiorespiratory function.

## 1. Introduction

Lung cancer is the leading cause of cancer-related death worldwide, contributing to 1.6 million deaths annually [1]. Surgical resection remains the best curative treatment option in patients with early-stage non-small cell lung cancer (NSCLC), and a patient’s preoperative status is important to assess the feasibility of undergoing surgical lung resection under general anesthesia. In particular, patients with poor pulmonary function and cardiorespiratory fitness are considered inoperable due to increased morbidity and mortality after surgical resection [2]. Both cardiopulmonary fitness and functional capacity are widely recognized as strong predictors of postoperative complications, specifically mortality and long-term survival, in NSCLC [3].

Cardiorespiratory fitness (CRF) and functional capacity are affected by physical activity (PA) [4]. Numerous studies have conducted PA or exercise programs to improve physical fitness and functions before thoracic surgery [2,5]. Adopting and sustaining a more physically active lifestyle has been shown to reduce the risk of complications and mortality and improve health-related quality of life in patients who underwent thoracic surgery [6]. However, promoting long-term PA has been challenging due to various factors such as lack of motivation, access to facilities for physical activities, and inclement weather [7]. Studies reported that seasonal weather conditions could promote or deter PA [8]. Furthermore, most studies were conducted with a small number of participants (<50) and only included limited populations such as children [9] or the elderly [10]. In addition, few quantitative assessments have been focused on seasonal variation in PA among preoperative lung cancer patients. Studies have attempted to measure daily PA with quantitative assessments using simple and non-expensive devices during the perioperative periods of lung cancer surgery [11,12,13]. They found that that daily walk distance predicted maximum oxygen consumption per minute in patients undergoing lung resection [11], and the time and the quality of the daily ambulatory activity of the patients decreased during the first postoperative month [12]. Thus, our study aims to use a wearable device (Fitbit) to examine how the season and temperature level affect PA among patients who are scheduled to undergo surgical resection for lung cancer.

## 2. Methods

### 2.1. Subjects and Data Sources

Patients with lung cancer in this study were selected from the Coordinate Approach to Cancer patients’ Health for Lung Cancer (CATCH-LUNG) cohort of preoperative lung cancer patients between March 2016 and October 2018 at the Samsung Medical Center in Seoul, Korea. Inclusion criteria for CATCH-LUNG cohort were (1) patients who were expected to undergo curative lung cancer surgery for suspected or histologically confirmed NSCLC, (2) patients who were able to walk and keep a normal life with the Eastern Cooperative Oncology Group Performance Status (ECOG PS ≤1), and (3) patients understood the purpose of this study and agreed to participate in the study. Exclusion criteria were (1) patients who had undergone neoadjuvant treatment before surgery, (2) patients free of NSCLC after pathological exams, (3) patients whose surgery was canceled, or (4) patients who withdrew consent before baseline data collection. Among patients who met these criteria, we furthermore excluded patients who had either pathologically confirmed stage IV cancer after surgery (*n* = 2) and 63 patients who were excluded due to lack of Fitbit data (28 patients wore their Fitbit less than one day and 35 patients had either hardware or software failures of Fitbit). The final study sample included 555 patients. The study protocol was approved by the Institutional Review Board of Samsung Medical Center (no. 2015-11-025). Written informed consent was obtained from all participants.

### 2.2. Grouping and Weather Data Collection

The main exposure variable was the season, which was divided into spring (March to May), summer (June to August), autumn (September to November), and winter (December to February) using the study enrollment date before surgery. For preoperative patients living in the metropolitan Seoul area, the weather-related factor of wind chill temperature was obtained from the Korea Meteorological Administration (https://data.kma.go.kr).

### 2.3. Physical Activity and other Variable

The main outcome was PA, which was assessed using a reliable wearable activity tracker [14]. The Flex tracker (Fitbit, San Francisco, CA, USA) was used to quantify the PA intensity, time, activity type, and steps per day. We asked participants to wear a Fitbit activity tracker 24 h per day for 7 consecutive days. We determined that patients did not wear the Fitbit if there was no movement (0 steps) for more than 4 consecutive hours during daytime (9:00 a.m. to 4:00 p.m.). Physical activity time, frequency, and intensity data were automatically measured and saved by an internal sensor. We then calculated activity level by combining the Fitbit data with age, gender, height, and weight data recorded at registration. Activities were classified into four categories: (1) sleeping or sedentary activity (1 MET), (2) light physical activity (1~2.9 METs), (3) moderate physical activity (3~5.9 METs), and (4) vigorous physical activity (more than 6 METs). The calculated values were averaged to define daily activity level.

The tracker was worn on the wrist. To reduce bias, the device had no screen so that patients could not see their recorded level of PA. In addition, there was no specific education or guidelines for patients regarding PA and patients were recommended to maintain PA prior to surgery, as usual.

CRF was measured using the 6-min walk test (6MWT), which was performed according to ATS guidelines [15]. Each participant was asked to walk (not run) back and forth along the corridor as far as possible for 6 min and was given standardized verbal encouragement every minute. The test has been widely used for preoperative and postoperative evaluations of CRF. In some clinical situations, the 6MWT provides a better index of the patient’s ability to perform daily activities compared to peak oxygen uptake [16]. In addition, the 6MWT is a sub-maximal test of CRF, as opposed to cardiopulmonary exercise testing (CPET). While it is well known that the incremental shuttle walk test (ISWT) has a higher correlation with CPET than 6MWT, we could not use the ISWT because it was not available in Korea. In fact, 6MWT has been widely used in real-world clinics for cardiopulmonary evaluation in patients with chronic obstructive pulmonary disease (COPD) [17]. To obtain quality data, trained researchers provided study participants detailed instructions about 6MWT, and asked patients to do a pilot walk (for 15~20 s) before the actual test.

Spirometry and DLco measurements were performed using a Vmax 22 respiratory analyzer (SensorMedics, OH, USA) according to the American Thoracic Society/European Respiratory Society criteria [18,19]. Absolute values of forced expiratory volume in 1 s (FEV_1_), forced vital capacity (FVC), and DLco were obtained, and the percentage of predicted values (% predicted) for FEV_1_, FVC, and DLco were calculated using a representative Korean sample [20,21] as a reference. Sociodemographic and behavioral information, including age, smoking status, and comorbidities, were recorded before surgery using a questionnaire. Treatment information regarding pathological stage and pulmonary function were collected after surgery.

### 2.4. Statistical Analysis

Continuous and categorical variables were compared among the seasons using analysis of variance (ANOVA) and the *χ2* test, respectively.

For the main analyses, we used linear regression to compare the daily number of steps and the moderate-to-vigorous physical activity (MVPA) time by season. Since steps per day and MVPA minutes are markedly right skewed (*p*-values based on Shapiro–Wilk and Shapiro–Francia tests for normality were <0.001), we used log-transformed daily number of steps and MVPA time as the outcomes. The average difference (as a percent difference with 95% confidence interval (CI)) was estimated comparing patients enrolled in the summer, autumn, or winter to those patients enrolled in the spring.

Using daily number of steps and MVPA duration, we developed a dichotomized outcome to evaluate the proportion of participants who were physically active. Being physically active was defined as either taking more than 8000 steps per day or performing more than 60 min of MVPA. We chose 8000 steps per day based on a reference that adults usually take 5000 steps per day and perform daily activities such as house errands, walking, or shopping [22]. We then added 3000 steps per day to account for 30 min of MVPA (10 min of MVPA is around 1000 steps [23,24]). From this, we equated 8000 steps to 60 min of MVPA.

Logistic regression was conducted to compare the odds of being physically active by season with adjustments made for age, sex, smoking status, FEV_1_% pred, any pulmonary comorbidities (COPD, asthma, or ILD), and any extra-pulmonary comorbidities (cardiovascular diseases or diabetes mellitus). In addition, we performed stratified analyses to evaluate differences in PA associated with the season in prespecified subgroups of age (<65 vs. ≥65 years) [25] and CRF (<500 vs. ≥500 m in 6MWD) [26].

To determine factors leading to the differences in PA according to season, we conducted an additional analysis for patients (*n* = 85) living in the Seoul metropolitan area because we considered the bias of area environment and topography in Korea. To find an association between weather and daily steps, we modeled wind chill temperature as a continuous variable using restricted cubic splines with knots at the 5th, 35th, 65th, and 95th percentiles of the sample distribution to provide a flexible estimate of the dose-response relationship between weather factors and daily steps.

For all analyses, a *p*-value of <0.05 was considered statistically significant. All analyses were performed using STATA software, version 14 (Stata Corp LP, College Station, TX, USA).

## 3. Results

The characteristics of 555 patients are described in Table 1. The mean (SD) age and daily steps of study participants were 61.1 (8.9) years and 10,603 (5200) (range, 425–32,143), respectively. Among the participants, Fitbit data from 30.8% (*n* = 171), 32.1% (*n* = 178), 17.8% (*n* = 99), and 19.3% (*n* = 107) of the study subjects were collected in the spring, summer, autumn, and winter seasons, respectively. Although patients in the winter season were likely to be younger and have better pulmonary function than patients in other seasons, the clinical characteristics, including sex, BMI, smoking status, comorbidities, pathologic stage, and cardiorespiratory fitness, were not different across the seasons (Table 1).

**Figure 1:**
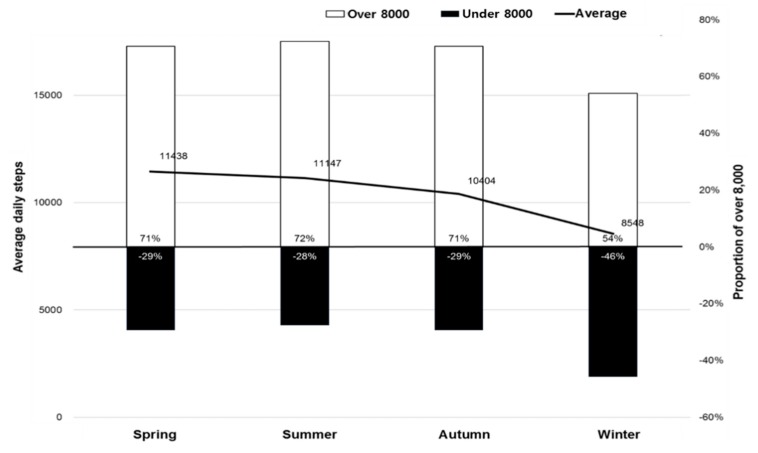
Mean daily steps and the proportion of participants who had more than 8000 steps in each season.

The mean (SD) daily steps were 11,438 (5922), 11,147 (5065), 10,404 (4403), and 8548 (4293) in patients who participated in this study in the spring, summer, autumn, and winter seasons, respectively. The proportion of patients who had 8000 or more steps per day was 71%, 72%, 71%, and 54% in participants who received surgery in the spring, summer, autumn, and winter seasons, respectively (Figure 1).

In the fully-adjusted models, patients in the winter season had a significantly lower number of daily steps, with a low of 27.04% (95% CI = −36.68%, −15.93%) compared to the daily steps of patients in the spring season. In comparing the mean (SD) MVPA time, patients in the winter season had the lowest MVPA among subjects (spring, 60.3 (57.2) min/d; summer, 58.5 (46.4) min/d; autumn, 51.0 (35.6) min/d; and winter, 35.0 (36.3) min/d). In the fully-adjusted models, compared to patients in the spring season, the MVPA time was significantly shorter by 35.22% (95% CI = −49.18%, −17.43%) for patients in the winter season. The number of steps and duration of MVPA were significantly lower in the winter season compared to spring season irrespective of age and 6MWD (Table 2).

In the fully-adjusted models, the OR for 8000 or more steps per day was 0.46 (95% CI 0.28, 0.77) for patients in the winter compared to those in the spring. The proportion of patients who had 60 min or more MVPA per day was also similar (spring, 30.4%; summer, 42.7%; autumn, 32.3%; winter, 18.7%). In the fully-adjusted models, the OR for MVPA ≥ 60 min/day was 0.52 (95% CI = 0.29, 0.94) for patients in the winter compared to those in the spring. In particular, the OR for low CRF (<500 m) was 0.30 (95% CI 0.10, 0.87) for patients in the winter compared to those in the spring (Table 3).

**Figure 2:**
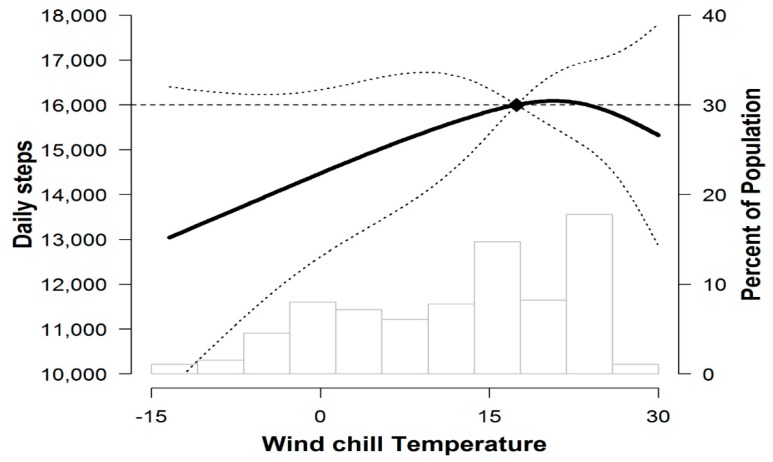
Mean daily steps (95% CI) by wind chill temperature. The curves represent daily step (solid line) and their 95% confidence intervals (dashed lines) based on restricted cubic splines for wind chill temperature with knots at the 5th, 35th, 65th, and 95th percentiles of their sample distributions. The reference value (diamond dot) was set at the 90th percentile.

In the spline regression models, the relationship between wind chill temperature and daily steps was linear below 25° wind chill temperature, with lower daily steps corresponding to colder temperatures (Figure 2). The association between winter and the decline in PA was consistent across all the subgroups.

## 4. Discussion

We found that preoperative PA was significantly lower in the winter season compared to other seasons, irrespective of age and CRF. In particular, the low MVPA in the winter season was more robust in patients who had low CRF, but the effect was not statistically significant. In terms of weather factors, wind chill temperature was inversely associated with PA. These results demonstrate that the PA of preoperative lung cancer patients is significantly affected by the season and temperature levels.

Our study showed that daily step was lower by 27% in the winter season compared with the spring season. These findings are consistent with previous studies that showed the association between daily PA and the season [8,27,28,29,30]. Those studies observed that PA decreased during the winter season in healthy adults [8] and elderly subjects [29]. This phenomenon was profoundly observed in patients with lung and heart diseases. In COPD patients, the daily step count reduced by 43.3 steps/day/°C in the winter season [27], and in heart failure patients, there was a significant seasonal variation in activity between summer and winter ranging from 13.78% to 20.69% [30]. In addition, the odds of walking more than 8000 steps or having more than 60 min MVPA per day in winter is around 0.5 compared to those in spring. It means that only about 50% of patients in the winter season had similar levels of PA as those of patients in spring time. It would be important for clinicians and researchers to evaluate PA in different seasons, especially in winter and provide more tailored educations for PA depending on season. In fact, the cold and snow of winter weather can significantly affect participation in outdoor activity. In particular, influenza during the winter season is an important contributor to the winter burden among older adults and lung-disease patients [31]. Furthermore, slippery roads and a high risk of falling can affect the decline in PA [32]. When outdoor activities are complicated by weather conditions, indoor activity equipment such as treadmills and stationary bicycles offer a helpful alternative. A recent study found that preoperative exercise was effective in reducing postoperative complications and length of hospital stay in patients with lung cancer [6], suggesting the importance of maintaining PA before lung cancer surgery. Another study found that that pulmonary rehabilitation programs using treadmills and bicycles were a valid preoperative strategy to improve physical performance in patients with both NSCLC and COPD [33]. It would be worthwhile to develop a PA intervention program for winter and evaluate its impact on postoperative pulmonary complications.

We found that a few patients achieving MVPA ≥ 60 min/day were more robust in patients with low CRF (≤500 m of 6MWD) compared to those with high CRF (>500 m of 6MWD). Lower exercise tolerance assessed through the 6MWD is associated with poorer postoperative outcomes after lung resection [34]. However, a 7-day intensive preoperative program of PA combined with inspiratory muscle training increased the 6MWD in lung cancer patients in a previous study [35]. Increasing PA has positive effects on CRF and muscle power, such as health-related physical function, leading to improved postoperative recovery in early-stage NSCLC patients. In a previous study, structured and planned preoperative PA reduced postoperative complications (from 45% to 67%) and the length of hospital stay (by 4–5 days) in patients with lung cancer [3,6], suggesting that preoperative PA is an effective strategy for lung cancer patients who will undergo surgical resection. Thus, a strategic PA program should be recommended to maintain and promote PA even in the winter season, especially for low-CRF patients and older adults, for whom it is feasible to receive surgery for lung cancer.

However, seasons are a crude measure when it comes to understanding the effects of weather on PA [36]. Previous studies have observed the effects of several factors related to the season on daily activities. In a healthy population, there was a 2.9% decrease in steps per day for every 10 °C drop in temperature [37]. In addition to temperature, day length and daylight hours account for 73% of the monthly differences in daily activity level, and these three parameters are independent predictors of daily activities [38]. To analyze the climate conditions affecting PA in our study, we conducted a subgroup analysis to identify the relationship between climate conditions and activity for 85 people living in the capital city of South Korea. We found that wind chill temperature significantly affected the steps per day of preoperative patients. In the spline regression models, the relationship between wind chill temperature and daily steps was linear below 25 °C wind chill temperature, with lower daily steps corresponding to colder temperatures. This is similar to the previous findings [30,39]. The wind chill temperature used in our study is likely to be more closely related to patients’ PA behavior compared to absolute temperature, as the wind chill temperature is a quantitative measure of the heat or cold that the human body feels, calculated based on air temperature and wind velocity [40]. While it is not possible to change the weather conditions, a better understanding of how weather influences PA might be helpful when developing strategies to ameliorate the impact of adverse weather conditions on future PA interventions in low-CRF patients and older adults.

There are several limitations to this study. Firstly, this was a cross-sectional study and we were not able to observe the change of PA of the same patient group in different seasons. In addition, due to enrollment periods, a limited number of patients were observed in the winter season. These factors limited our ability to observe the magnitude of seasonal variation in PA among patients. Despite these limitations, our study indicates little variability in PA by season. In fact, patients in the winter season were younger and had better lung function than patients in other season and we still see the different levels of PA depending on season. Secondly, patients who were motivated to perform PA might be more likely to participate in the study. The mean daily step figures were higher in this study compared to data reported in earlier studies [11]. This might be due to our recruitment of patients with ECOG 0 or 1 as they would be healthier and more physically active than general cancer patients. However, mean 6MWD was similar to that reported in the previous study [26], suggesting our study participants had similar CRF to other lung cancer patients. Thirdly, the physical activity device used only counts the steps and the time spent on all those steps, but it does not differentiate whether the steps are outside or inside the house. Therefore we have a limitation in assuming that patients in the winter season were less active due to decreased outdoor PA. Lastly, the results of this study might not be generalizable as it was conducted with patients at a tertiary cancer center in Seoul, Korea. Additional studies would be necessary to confirm the findings with different populations and in different settings.

## 5. Conclusions

In this study, we found that season and temperature levels affect PA among preoperative lung cancer patients. Patients were much less physically active in the winter season than other seasons and patients in the winter season had lower cardiorespiratory function. Health professionals need to be aware of these seasonal differences and recommend indoor physical activities that preoperative patients with lung cancer can do in winter.

## Figures and Tables

**Table 1:** Characteristics of study participants by season (*n* = 555).

Characteristics	Spring (*n* = 171)	Summer (*n* = 178)	Autumn (*n* = 99)	Winter (*n* = 107)	*p*
**Mean age**	60.6 (8.9)	62.6 (8.3)	60.6 (9.0)	59.9 (9.4)	0.05
**Age categories**					0.04
<65	118 (69.0)	104 (58.4)	66 (66.7)	79 (73.8)	
≥65	53 (31.0)	74 (41.6)	33 (33.3)	28 (26.2)	
**Sex, male**	97 (56.7)	101 (56.7)	57 (57.6)	57 (53.3)	0.92
**Body mass index, kg/m^2^**	24.1 (2.9)	24.3 (2.7)	24.1 (2.7)	24.5 (3.2)	0.67
**Smoking status**					0.32
Never-smoker	78 (45.6)	89 (50.0)	48 (48.5)	57 (53.3)	
Ex-smoker	59 (34.5)	34 (19.1)	27 (27.3)	16 (15.0)	
Current smoker	34 (19.9)	55 (30.9)	24 (24.2)	34 (31.8)	
**Marital status**					0.64
Married	151 (88.3)	159 (89.3)	91 (91.9)	91 (85.1)	
Single/divorced/widowed	19 (11.1)	19 (10.7)	7 (7.1)	15 (14.0)	
Unknown	1 (0.6)	0	1 (1.0)	1 (0.9)	
**Employment status**					0.46
Current work	98 (57.3)	81 (45.5)	49 (49.5)	58 (54.2)	
No work	72 (42.1)	96 (53.9)	49 (49.5)	48 (44.9)	
Unknown	1 (0.6)	1 (0.6)	1 (1.0)	1 (0.9)	
**Monthly family income**					0.19
<$3.000	54 (31.6)	51 (28.7)	23 (23.2)	31 (29.0)	
≥$3.000	87 (50.9)	93 (52.3)	54 (54.6)	66 (61.7)	
Unknown	30 (17.5)	34 (19.1)	22 (22.2)	10 (9.4)	
**Comorbidities**					
**Pulmonary comorbidities**					
COPD	40 (23.4)	43 (24.2)	26 (26.3)	19 (17.8)	0.49
Asthma	3 (1.8)	8 (4.5)	1 (1.0)	3 (2.8)	0.34
ILD	1 (0.6)	2 (1.1)	1 (1.0)	2 (1.9)	0.86
**Extra-pulmonary comorbidities**					
Hypertension	52 (30.4)	72 (40.5)	28 (28.3)	35 (32.7)	0.12
Diabetes mellitus	22 (12.9)	27 (15.2)	11 (11.1)	7 (6.5)	0.18
Cardiovascular disease	12 (7.0)	20 (11.2)	9 (9.1)	9 (8.4)	0.58
**Pathologic stage**					0.81
I	128 (74.9)	130 (73.0)	75 (75.8)	75 (70.1)	
II	25 (14.6)	31 (17.4)	13 (13.1)	16 (15.0)	
III	18 (10.5)	17 (9.6)	11 (11.1)	16 (15.0)	
**Pulmonary function test**					
FVC, L	3.6 (0.9)	3.4 (0.8)	3.7 (0.9)	3.7 (0.8)	0.02
FVC, % predicted	93.4 (11.5)	89.8 (12.9)	94.3 (12.7)	96.4 (12.1)	< 0.01
FEV_1_, L	2.7 (0.7)	2.5 (0.6)	2.7 (0.6)	2.8 (0.6)	< 0.01
FEV_1_, % predicted	90.0 (13.2)	87.5 (15.5)	90.2 (14.9)	95.0 (13.5)	< 0.01
FEV_1_/FVC	73.8 (8.2)	73.6 (8.6)	72.6 (8.6)	75.1 (8.3)	0.21
DLco, %	91.6 (16.0)	89.2 (17.3)	89.7 (15.2)	91.3 (13.7)	0.47
**Cardiorespiratory fitness**					
6 min walk distance (m)	520.1(85.7)	506.9(89.1)	515.0(66.8)	508.1(80.7)	0.46
6 min walk distance					0.69
Short distance (<500 m)	68 (39.9)	77 (43.3)	37 (37.4)	49 (45.8)	
Long distance (≥500 m)	102 (59.7)	97 (54.5)	60 (60.1)	56 (52.3)	
Unknown	1 (0.6)	4 (2.3)	2 (2.1)	2 (1.9)	

Values are presented as either n (%) or mean (SD). COPD, chronic obstructive pulmonary disease; ILD, interstitial lung disease; FVC, forced vital capacity; FEV_1_, forced expiratory volume in 1 s; DLco, diffusing capacity of carbon monoxide.

**Table 2:** Differences in physical activity by season.

	Spring	Summer	Autumn	Winter
**Difference in steps per day (%)**				
**Overall**	Reference	−0.86 (−12.38, 12.18)	−6.24 (−18.86, 8.33)	−27.04 (−36.68, −15.93)
**Age**				
<65 years	Reference	−8.08 (−21.25, 7.30)	−10.27 (−24.74, 6.98)	−25.21 (−36.65, −11.70)
≥65 years	Reference	11.91 (−9.08, 37.74)	2.36 (−20.65, 32.02)	−32.16 (−48.31, −10.97)
*p* for interaction		0.14	0.40	0.55
**Cardiorespiratory fitness (6MWD)**				
<500 m	Reference	−1.21 (−18.1, 19.18)	7.07 (−14.97, 34.83)	−27.63 (−41.48, −10.50)
≥500 m	Reference	−0.75 (−15.53, 16.62)	−12.95 (−27.52, 4.55)	−20.97 (−34.54, −4.59)
*p* for interaction		0.97	0.17	0.54
**Difference in MVPA minutes per day (%)**				
**Overall**	Reference	5.06 (−14.6, 29.24)	−2.11(−23.13, 24.67)	−35.22 (−49.18, −17.43)
**Age**				
<65 years	Reference	5.13 (−19.26, 36.89)	−1.43 (−26.71, 32.57)	−33.03 (−49.63, −10.95)
≥65 years	Reference	4.05 (−26.37, 47.04)	−3.75 (−37.24, 47.60)	−40.72 (−62.76, −5.62)
*p* for interaction		0.96	0.93	0.66
**Cardiorespiratory fitness (6MWD)**				
<500 m	Reference	−4.25 (−30.56, 32.04)	20.57 (−18.5, 78.39)	−38.65 (−57.62, −11.18)
≥500 m	Reference	11.12 (−15.17, 45.55)	−10.57 (−34.3, 21.73)	−26.01 (−46.46, 2.25)
*p* for interaction		0.48	0.24	0.03

Models were adjusted for age, sex, smoking status, FEV_1_% pred, any pulmonary comorbidities, and any extra-pulmonary comorbidities. Pulmonary comorbidities include chronic obstructive pulmonary disease, asthma, or interstitial lung disease, and extra-pulmonary comorbidities include cardiovascular diseases or diabetes mellitus. 6MWD, 6-min walk distance. MVPA, moderate-to-vigorous physical activity.

**Table 3:** Odds ratios (95% CI) for physical activity by season.

	Spring	Summer	Autumn	Winter
**Steps ≥8000/day**				
**Overall**	Reference	1.11 (0.69, 1.79)	0.98 (0.57, 1.70)	0.46 (0.28, 0.77)
**Age**				
<65 years	Reference	0.79 (0.43, 1.44)	0.73 (0.37, 1.45)	0.38 (0.20, 0.71)
≥65 years	Reference	2.00 (0.93, 4.31)	1.75 (0.67, 4.56)	0.67 (0.26, 1.74)
*p* for interaction		0.06	0.15	0.32
**Cardiorespiratory fitness (6MWD)**				
<500 m	Reference	0.94 (0.48, 1.82)	1.75 (0.73, 4.20)	0.45 (0.21, 0.96)
≥500 m	Reference	1.35 (0.67, 2.74)	0.74 (0.36, 1.55)	0.58 (0.28, 1.22)
*p* for interaction		0.46	0.14	0.63
**MVPA ≥60 min/day**				
**Overall**	Reference	1.73 (1.10, 2.71)	1.07 (0.62, 1.83)	0.52 (0.29, 0.94)
**Age**				
<65 years	Reference	1.59 (0.91, 2.79)	1.19 (0.62, 2.27)	0.59 (0.30, 1.15)
≥65 years	Reference	2.11 (0.96, 4.61)	0.92 (0.34, 2.50)	0.29 (0.07, 1.13)
P for interaction		0.50	0.72	0.42
**Cardiorespiratory fitness (6MWD)**				
<500 m	Reference	1.26 (0.61, 2.58)	1.24 (0.52, 2.98)	0.30 (0.10, 0.87)
≥500 m	Reference	1.97 (1.10, 3.55)	1.03 (0.52, 2.05)	0.77 (0.37, 1.61)
*p* for interaction		0.34	0.75	0.15

Models were adjusted for age, sex, smoking status, FEV_1_% pred, any pulmonary comorbidities, and any extra-pulmonary comorbidities. Pulmonary comorbidities include chronic obstructive pulmonary disease, asthma, or interstitial lung disease, and extra-pulmonary comorbidities include cardiovascular diseases or diabetes mellitus. 6MWD, 6-min walk distance. MVPA, moderate-to-vigorous physical activity.
